# Isolation and Analysis of Anthocyanin Pathway Genes from *Ribes* Genus Reveals MYB Gene with Potent Anthocyanin-Inducing Capabilities

**DOI:** 10.3390/plants9091078

**Published:** 2020-08-22

**Authors:** Pavel Starkevič, Aušra Ražanskienė, Urtė Starkevič, Vaiva Kazanavičiūtė, Erna Denkovskienė, Vidmantas Bendokas, Tadeušas Šikšnianas, Rytis Rugienius, Vidmantas Stanys, Raimundas Ražanskas

**Affiliations:** 1 Department of Eukaryotic Gene Engineering, Institute of Biotechnology, Vilnius University, 10257 Vilnius, Lithuania; pavel.visarcuk@gmail.com (P.S.); ausra.razanskiene@bti.vu.lt (A.R.); star.urte@gmail.com (U.S.); kaaiva@yahoo.de (V.K.); erna@nomadsbio.lt (E.D.); 2 Nature Research Centre, Akademijos str. 2, 08412 Vilnius, Lithuania; 3 Department of Orchard Plant Genetics and Biotechnology, Institute of Horticulture, Lithuanian Research Centre for Agriculture and Forestry, 54333 Babtai, Lithuania; v.bendokas@lsdi.lt (V.B.); t.siksnianas@lsdi.lt (T.Š.); r.rugienius@lsdi.lt (R.R.); v.stanys@lsdi.lt (V.S.)

**Keywords:** anthocyanins, *Ribes nigrum*, *Ribes uva-crispa*, *Ribes rubrum*, MYB10, bHLH

## Abstract

Horticultural crops of the *Ribes* genus are valued for their anthocyanin-rich fruits, but until now, there were no data about the genes and regulation of their flavonoid pathway. In this study, the coding sequences of flavonoid pathway enzymes and their putative regulators MYB10, bHLH3 and WD40 were isolated, and their expression analyzed in fruits with varying anthocyanin levels from different cultivars of four species belonging to the *Ribes* genus. Transcription levels of anthocyanin synthesis enzymes and the regulatory gene *RrMYB10* correlated with fruit coloration and anthocyanin quantities of different *Ribes* cultivars. Regulatory genes were tested for the ability to modulate anthocyanin biosynthesis during transient expression in the leaves of two *Nicotiana* species and to activate *Prunus avium* promoters of late anthocyanin biosynthesis genes in *N. tabacum*. Functional tests showed a strong capability of *RrMyb10* to induce anthocyanin synthesis in a heterologous system, even without the concurrent expression of any heterologous bHLH, whereas *RrbHLH3* enhanced MYB-induced anthocyanin synthesis. Data obtained in this work facilitate further analysis of the anthocyanin synthesis pathway in key *Ribes* species, and potent anthocyanin inducer *RrMyb10* can be used to manipulate anthocyanin expression in heterologous systems.

## 1. Introduction

*Ribes*, the only genus of the Grossulariceae family, contains woody perennial plants native throughout temperate regions of the Northern Hemisphere. The first members of the genus were domesticated 400–500 years ago, and today descendants of at least eighteen species are widely cultivated in Europe and, to a smaller degree, in China, New Zealand and North and South America [1]. The most commercially important species are blackcurrant (*Ribes nigrum* L.) and redcurrant (*Ribes rubrum* L.). Other domesticated currants and gooseberries are commonly cultivated in home gardens, but also have some commercial value. According to the Food and Agriculture Organization of the United Nations (http://www.fao.org/faostat/en/#data/QC), the annual worldwide production of currants in 2018 was about 660,000 tons (almost all in Europe, nearly 90% in Eastern Europe), and the production of gooseberries surpassed 170,000 tons (almost all in Europe). Small fleshy fruits produced by numerous cultivars of the genus, especially blackcurrants, are considered an important source of health-promoting phytochemicals, possessing immunomodulatory, anti-inflammatory, antimicrobial and cancer prevention potential [2,3]. These valuable properties of blackcurrants are attributed mainly to high quantities of vitamin C (reaching over 350 mg/100 g fresh weight) and phenolic compounds, mainly anthocyanins (reaching over 500 mg/100 g fresh weight) [2,4,5]. Besides possessing strong antioxidant and other health-promoting qualities, anthocyanins are also the main components determining the color of berries, an important commercial feature [3,6].

**Figure 1:**
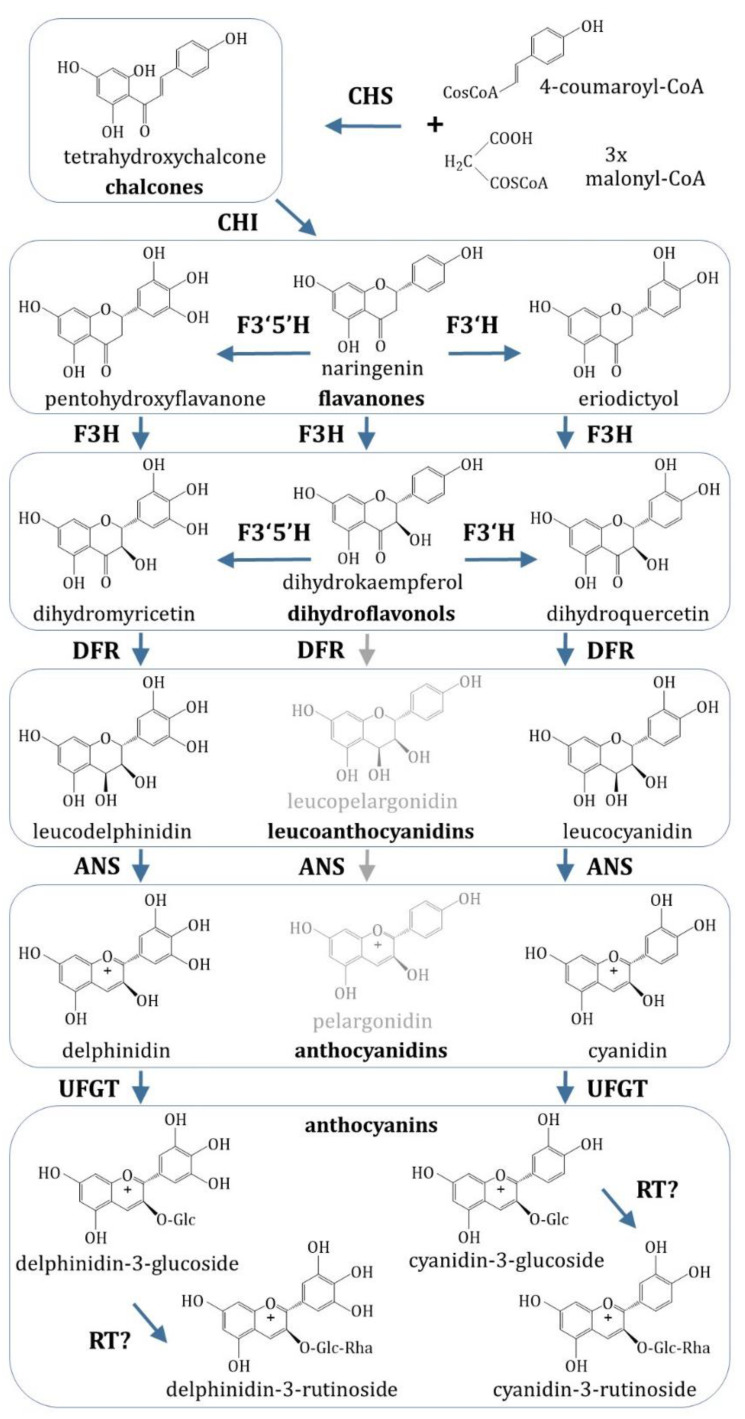
Putative anthocyanin pathway in fruits of the *Ribes* genus. Only the branches leading to the most prevalent *Ribes* anthocyanins, delphinidin-3-O-rutinoside and cyanidin-3-O-rutinoside, are completely shown. Abbreviations of enzymes: CHS, chalcone synthase; CHI, chalcone isomerase; F3H, flavonoid 3-hydroxylase; F3′H, flavonoid 3′-hydroxylase; F3′5′H, flavonoid 3’,5’-hydroxylase; DFR, dihydroflavonol reductase; ANS, anthocyanidin synthase; UFGT, UDP-glucose:flavonoid 3-O-glucosyl transferase; RT, putative rhamnosyltransferase.

More than 600 different anthocyanin structures are known, and at least fifteen of them have been found in blackcurrant [7]. Several studies aimed to determine the composition and quantities of anthocyanins in different cultivars of currants and gooseberries and revealed the predominance of cyanidin-3-O-rutinoside, delphinidin-3-O-rutinoside, cyanidin-3-O-glucoside and delphinidin-3-O-glucoside [4,8,9]. The key intermediates and enzymes leading to the production of different anthocyanin classes are already well investigated in a model plant, *Arabidopsis thaliana*, and many crop species [10,11,12]. The spectrum of anthocyanin molecules synthesized in *Ribes* fruits suggests a scheme of an anthocyanin synthesis pathway with an inactive pelargonidin branch (Figure 1), which is a subset of the general flavonoid pathway [13].

The main regulators of anthocyanin synthesis in different plant tissues are MYB R2R3 class transcription factors, which act in concert with bHLH and WD40 proteins by forming the so-called MBW complex [14]. Based on gene expression studies of *Arabidopsis* and some other plants, the flavonoid biosynthetic pathway is subdivided into “early” (chalcone synthase (CHS), chalcone isomerase (CHI), flavanone 3-hydroxylase (F3H)) and “late” (dihydroflavonol 4-reductase (DFR), anthocyanin synthase (ANS), UDP-glucose:flavonoid glucosyltransferase (UFGT)) genes. It was established that the MBW complex regulates the expression of late genes, but the presence of this complex is not required for the expression of early genes. However, the exact late and early sets differ between species and even tissues, and they are determined by interactions between different MYB and bHLH proteins, while plentiful MYB variants are responsible for the fine-tuning of pigment production [15]. Recently, the expressions of the anthocyanin biosynthetic pathway and related genes have been studied through global transcriptome methods, uncovering a wealth of information in important fruit crops, such as sweet cherry, bayberry and black raspberry [16,17,18]. These studies confirmed that anthocyanin production correlates with the expression levels of the main structural genes and some MYB proteins, although the presence of early and late gene sets was not always discernible.

Despite the importance of anthocyanins and intensive studies of their composition and properties, until now, there were no data about flavonoid pathway genes and their regulation in berries of the *Ribes* genus. The whole Saxifragales clade is not studied at the molecular level as extensively as, for example, the asterid and rosid clades, no genomes are sequenced and not much overall sequence information is available. The only exception now is the genus *Paeonia*, with the expression of anthocyanin pathway genes analyzed in *P. lactiflora* and *P. suffruticosa* flowers [19]. However, anthocyanin content in *Paeonia* flowers differs significantly from that of *Ribes* fruits, with peonidin, pelargonidin and cyanidin 3- and 3,5-glucosides being the main species in *Paeonia* [20]. Although anthocyanin-regulating MYBs and, to a lesser degree, the other regulators have been analyzed in many plants, such genes in Saxifragales were only recently identified in *P. suffruticosa* [21,22,23].

In the present study, our aim was to isolate anthocyanin pathway genes and their regulators, to analyze their expression in fruits of different *Ribes* species and their cultivars to identify ones associated with anthocyanin levels and to test the anthocyanin-inducing capabilities of isolated transcription factors in a heterologous expression system.

## 2. Results

### 2.1. Isolation of Anthocyanin Pathway Genes from Four Ribes Species

A PCR with a degenerated primers approach was used to clone genomic and cDNA fragments from different *Ribes* species. Since the genus *Ribes* belongs to the Saxifragales taxon, for which no sequence information on flavonoid pathway genes was available at the start of this work, the primers were designed to target most conservative regions in all known relevant gene sequences, mainly from the rosid and asterid taxa. The sequences were cloned from mature fruits of cultivars with the highest fruit anthocyanin content: *R. nigrum* “Ben Tirran”, *R. uva-crispa* “Chorny negus”, *R. rubrum* “Jonkheer van Tets” and *R. aureum* “Cordona’”. After sequence information for the *Ribes* gene fragment was obtained, 3′- and 5′-RACE (rapid amplification of cDNA ends) was applied to clone full or partial sequences of the main anthocyanin pathway enzymes UFGT, ANS, DFR, F3H, F3′H, F3′5′H, CHI and CHS, as well as transcriptional regulators MYB10, bHLH3 and WD40. To gain the sequence information needed to design *Ribes* cross-species qPCR primers, 200–300 bp fragments were also sequenced from other analyzed cultivars.

Sequence comparisons revealed that enzymes of different *Ribes* species share close homology (>90% of identical residues) at both the amino acid and nucleotide level. Noncoding flanking sequences differ much more and have insertions–deletions. The similarity between *Ribes* and *Paeonia* for some enzymes (ANS, F3H) is 83–88% of identical residues at the amino acid level and 77–81% at the nucleotide level, whereas the similarity of *Ribes* sequences to enzymes of species belonging to the rosid, asterid and caryophyllales clades is generally lower. Meanwhile, the similarity between other *Ribes* and *Paeonia* enzymes is not as high, probably because different variants of the same enzymes are expressed in *Ribes* fruits and *Paeonia* petals, the sources of the analyzed sequences (Figures S1–S9).

**Figure 2:**
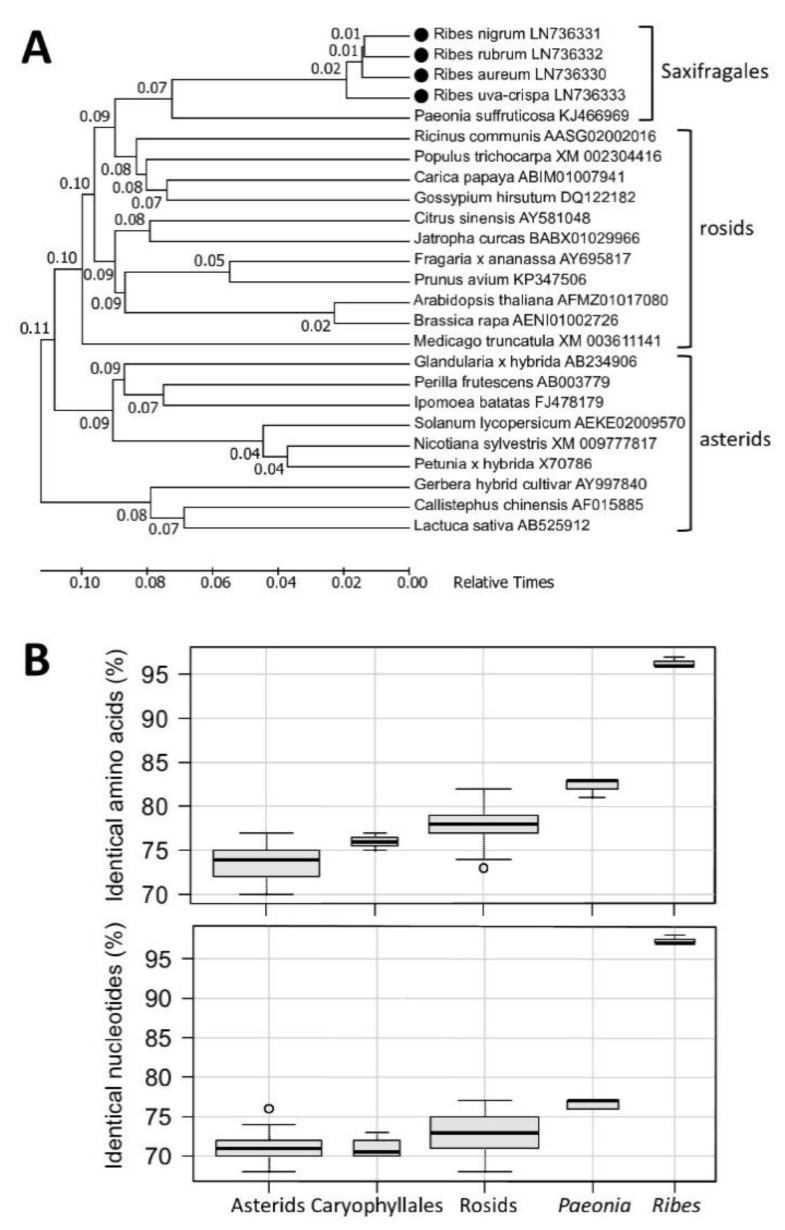
Similarity between ANS sequences from *Ribes* genus and other taxa. (**A**) A phylogenetic timetree of selected ANS protein sequences. Divergence times were calculated with the RelTime method using MEGA7. All proteins are named with the species name and accession number. (**B**) Boxplots showing the similarity between ANS sequences. Each boxplot shows the spread of percentages of identical residues in pairwise alignments between *R. nigrum* ANS and other ANS sequences. In each group (except *Ribes* and *Paeonia*) each sequence aligned to *R. nigrum* ANS represents a different genus. The number of pairwise alignments in each group: asterids, 21; caryophyllales, 4; rosids, 22; *Paeonia*, 5; *Ribes*, 4.

The similarity of *Ribes* flavonoid pathway enzymes to the counterparts from other species can be best exemplified by comparing ANS sequences (Figure 2). Sequence information for ANS is available from many species and, based on the available sequence data, it can be hypothesized that many plant species possess only one functional ANS gene per haploid genome [16,17,18]. This hypothesis is indirectly supported by the fact that ANS mutants, such as white pomegranate and yellow raspberry cultivars, as well as an *A. thaliana* mutant, do not synthesize anthocyanins in any tissues, in any stage of development or in response to any environmental factors [24,25,26]. As can be seen in the phylogenetic timetree (Figure 2A), *Ribes* ANS protein sequences are more homologous to the sequences of rosids than to the asterid clade, and sequences inside the asterid branch are more divergent, although that is not true for sequences of all anthocyanin pathway enzymes. More sequence comparisons with percentages of identical amino acids and nucleotides to *R. nigrum* ANS are depicted by boxplots (Figure 2B) and the phylogenetic trees of main pathway enzymes are presented in supplementary figures.

Anthocyanin pathway genes are regulated by a ternary complex MBW, which includes WD40 and proteins from the bHLH and MYB families. In this work, we tried to clone all three members of this regulatory complex. The sequence of the only constant member of this complex, WD40, is highly conservative, and this was also confirmed with *Ribes*. The *R. nigrum* WD40 protein shares at least 75% identical residues with any known counterpart from the asterid and rosid clades (Figure S7).

### 2.2. Anthocyanin-Regulating Genes of Ribes Genus

**Figure 3:**
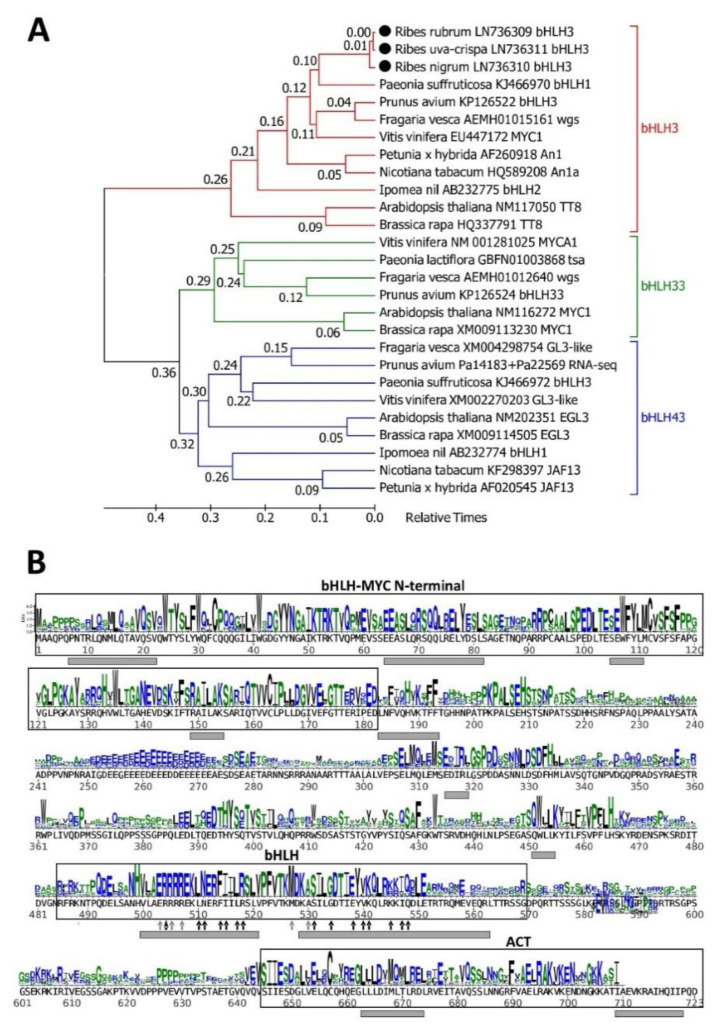
*Ribes rubrum* bHLH3 is homologous to other bHLH3-TT8 proteins. (**A**) A phylogenetic timetree of selected bHLH sub-group IIIf protein sequences. All proteins named with species name, accession number and original protein name. Sources of unnamed sequences: wgs, genes identified from NCBI Whole Genome Sequencing database; tsa, sequences identified from NCBI Transcriptome Shotgun Assembly sequence database; RNA-seq, cDNA from whole transcriptome study. (**B**) Sequence logo representing an alignment of 80 bHLH3-like proteins from different species of the Pentapetalae taxonomic branch. Empty boxes denote protein domains as defined in NCBI’s Conserved Domain Database. Gray boxes beneath the sequence specify helixes as predicted by PSIPRED. Gray arrows point to amino acids involved in DNA binding; black arrows point to amino acids involved in the dimerization interface; black arrow with long head points to amino acid essential to E-box specificity.

Since the bHLH3 protein is the most obvious activating bHLH member of the MBW complex, it was cloned from the *Ribes* genus in this work. As can be seen in the phylogenetic timetree (Figure 3A), the cloned sequence from *R. rubrum* clearly belongs to the bHLH3/TT8 branch of bHLH group IIIf proteins. Proteins belonging to different Saxifragales genera are positioned inside the rosid clade according to the created phylogenetic tree. The sequence logo with marked structural elements and known important amino acids (Figure 3B) shows that the cloned cDNA encodes all features needed to make functionally active bHLH3 protein.

**Figure 4:**
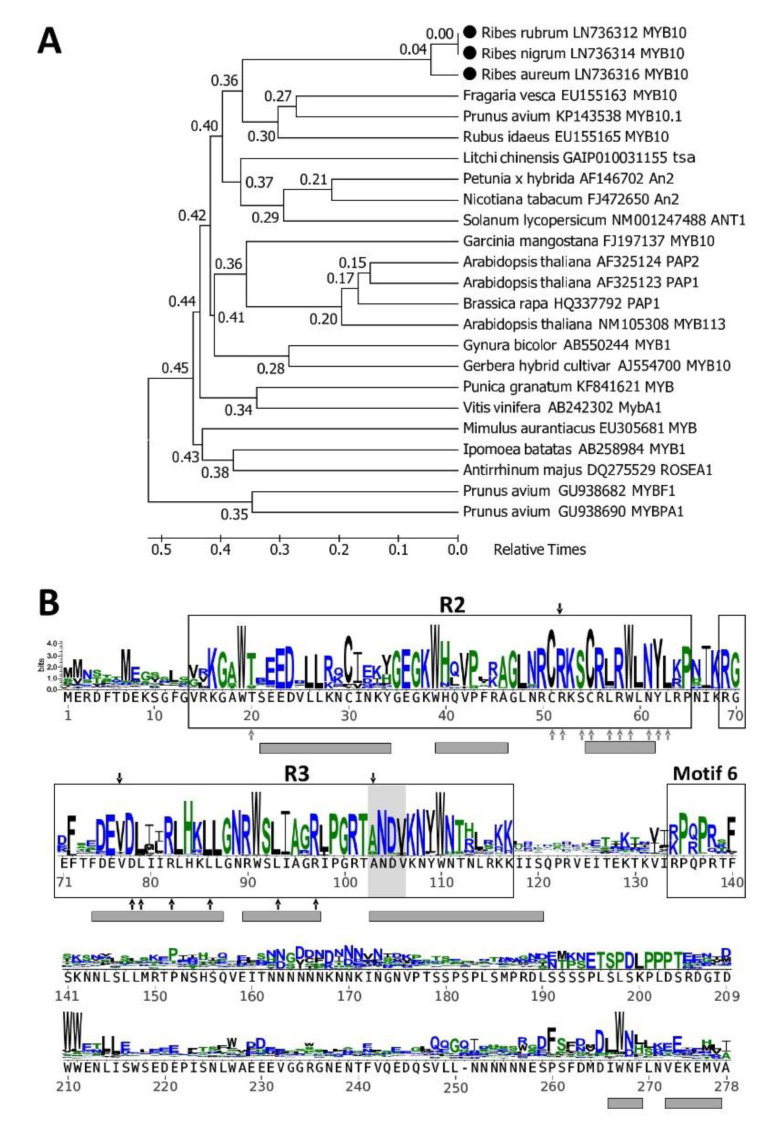
Similarity between *Ribes* MYB10 and other anthocyanin-inducing MYB proteins. (**A**) A phylogenetic timetree of selected anthocyanin-inducing MYB (MYB10-like) proteins. MYBF1 and MYBPA1 are non-MYB10-like, they activate flavanol- and proanthocyanidin-synthesizing enzymes, respectively. All proteins are named exactly the same way as described in the Figure 3 legend. (**B**) Sequence logo representing an alignment of 117 MYB10-like proteins from different species of the Pentapetalae taxonomic branch. Motif 6 (originally defined as KPRPR[S/T]F) is specific to subgroup 6 of the MYB proteins [27]. Gray background and black arrows on the top of the sequence denote amino acids specific to anthocyanin regulators [28]. Gray arrows point to amino acids important to DNA binding [29]. Black arrows under the sequence point to the motif ([DE]Lx2[RK]x3Lx6Lx3R) involved in interaction with bHLH proteins [30]. Other annotations are the same as described in the Figure 3 legend.

The last member of the MBW complex is a protein from the ubiquitous MYB family. In this work, we aimed to clone the gene encoding R2R3 class subgroup 6 protein, homologous to the group named MYB10 in Rosaceae and some other species. A phylogenetic tree composed of arbitrary selected subgroup 6 MYB proteins (Figure 4A) places *Ribes* sequences together with other anthocyanin-regulating proteins, but not with MYB proteins regulating other branches of the flavonoid pathway. The sequence logo (Figure 4B) shows that all important functional and structural elements of anthocyanin-regulating MYB proteins are present in the *Ribes* MYB10 sequences, and the presence of motif 6 places them in subgroup 6 of the MYB superfamily.

### 2.3. Expression of Anthocyanin Pathway Genes in Fruits of Different Ribes Cultivars

**Figure 5:**
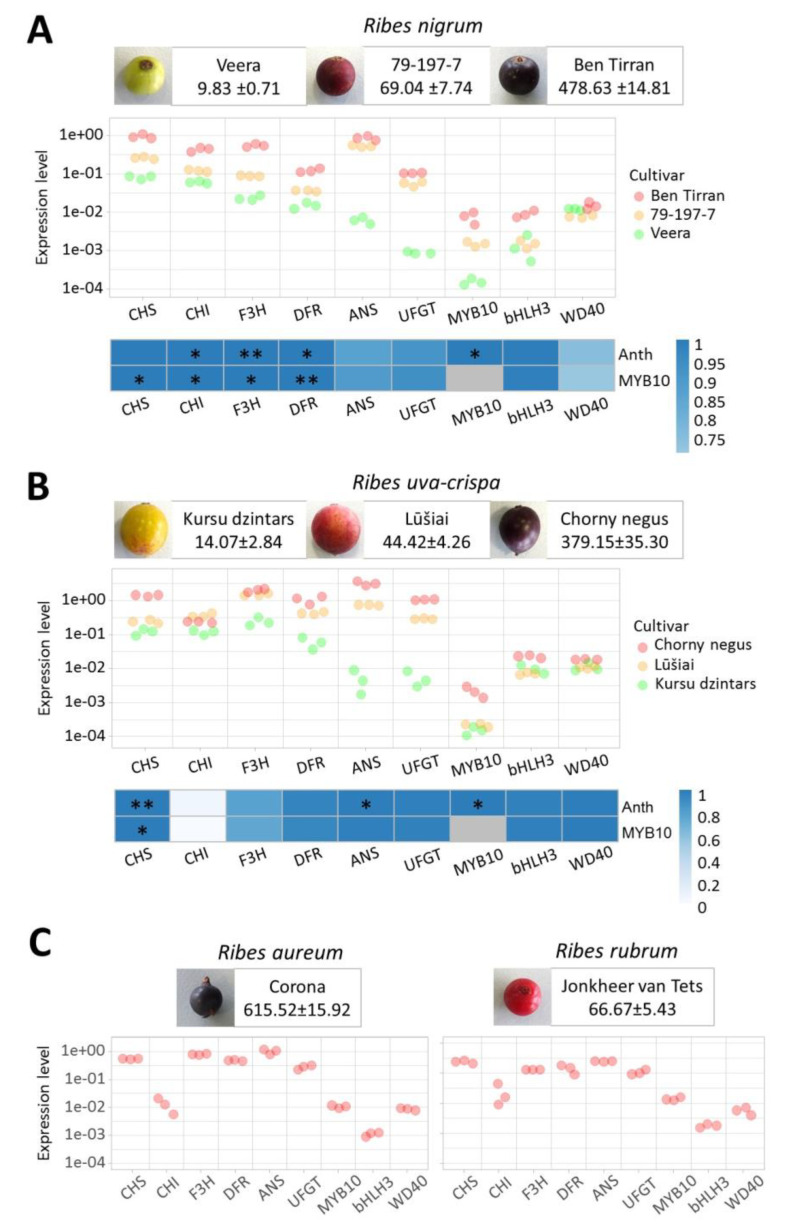
Expression of flavonoid pathway genes in fruits of different *Ribes* cultivars. For each species, ripe fruits with specified total anthocyanin content in mg/100 g of fresh fruit weight are shown at the top, qPCR expression data in the middle and Pearson correlation analysis of expression levels of each gene and anthocyanin content (Anth) or MYB10 expression level at the bottom. Each point in the gene expression dot plots represents the average of three technical replicates. All expression data are presented on a logarithmic scale and normalized by the level of control gene expression. The statistical significance of Pearson correlation is denoted as: *, *p* value ≤ 0.05; **, *p* value ≤ 0.01. (**A**) Three *Ribes nigrum* cultivars with different anthocyanin levels. (**B**) Three *Ribes uva-crispa* cultivars with different anthocyanin levels. (**C**) *Ribes rubrum* cultivar “Jonkheer van Tets” and *Ribes aureum* cultivar “Corona”.

To evaluate the expression of anthocyanin pathway genes, we designed oligonucleotide primer pairs targeting regions of genes for which sequence information was available for all analyzed species. Gene expression analysis was performed by qPCR using the same gene-specific primer pair used to analyze different species of the *Ribes* genus. To test whether cloned genes were differentially expressed in fruits with different anthocyanin contents, three cultivars containing high, medium or low quantities of fruit anthocyanins were selected from *R. nigrum* (Figure 5A) and *R. uva-crispa* (Figure 5B) species. One cultivar with high fruit anthocyanin content was selected from *R. rubrum* and R. *aureum* (Figure 5C) to verify whether the designed oligonucleotide primers are suitable for the analysis of these *Ribes* species.

Gene expression assay by qPCR showed that all genes encoding anthocyanin pathway enzymes were expressed at higher levels in fruits of the same species with higher anthocyanin levels (Figure 5A,B). Expression levels of genes encoding enzymes participating in the final stages of the anthocyanin pathway differed the most markedly. For example, expression levels of the *ANS* and *UFGT* genes differed by more than two to almost three levels of magnitude between cultivars with the highest and lowest anthocyanin content.

The expression levels of putative regulatory genes were generally lower. The expression level of the *WD40* gene was almost constant in different species and cultivars with different anthocyanin levels. The expression of the *bHLH3* gene was more variable between different species and different cultivars. However, there was no clear difference in the expression levels of *bHLH3* between cultivars with lower anthocyanin content in both *R. nigrum* and *R. uva-crispa*. The relative expression levels of the *MYB10* gene were not high in cultivars of *R. nigrum* and especially in cultivars of *R. uva-crispa*. Nevertheless, the expression of the *MYB10* gene is obviously higher in cultivars with higher anthocyanin levels, and this can be seen more clearly in the case of *R. nigrum* (Figure 5A).

Pearson correlation analysis revealed positive correlation between the expression of almost all genes and the anthocyanin content, as well as with the expression of *MYB10*. However, although the highest differences between cultivars with lowest ant highest anthocyanin content can be seen in the expression of the *ANS* and *UFGT* genes, the statistical significance of correlation between the expression of these genes and anthocyanin content (or expression of *MYB10*) is not as high as for some other genes. This can be explained by the observation that the expression patterns of these two genes in different cultivars do not follow the patterns observed for the anthocyanin content or *MYB10* expression. The highest difference in expression of *ANS* and *UFGT* is between cultivars with lower anthocyanin content, whereas the highest difference in anthocyanin content (and expression of *MYB10*) is between cultivars with higher anthocyanin content.

### 2.4. Transient Expression of Ribes rubrum MYB10 and bHLH3 Regulatory Genes in Leaves of Nicotiana Plants

**Figure 6:**
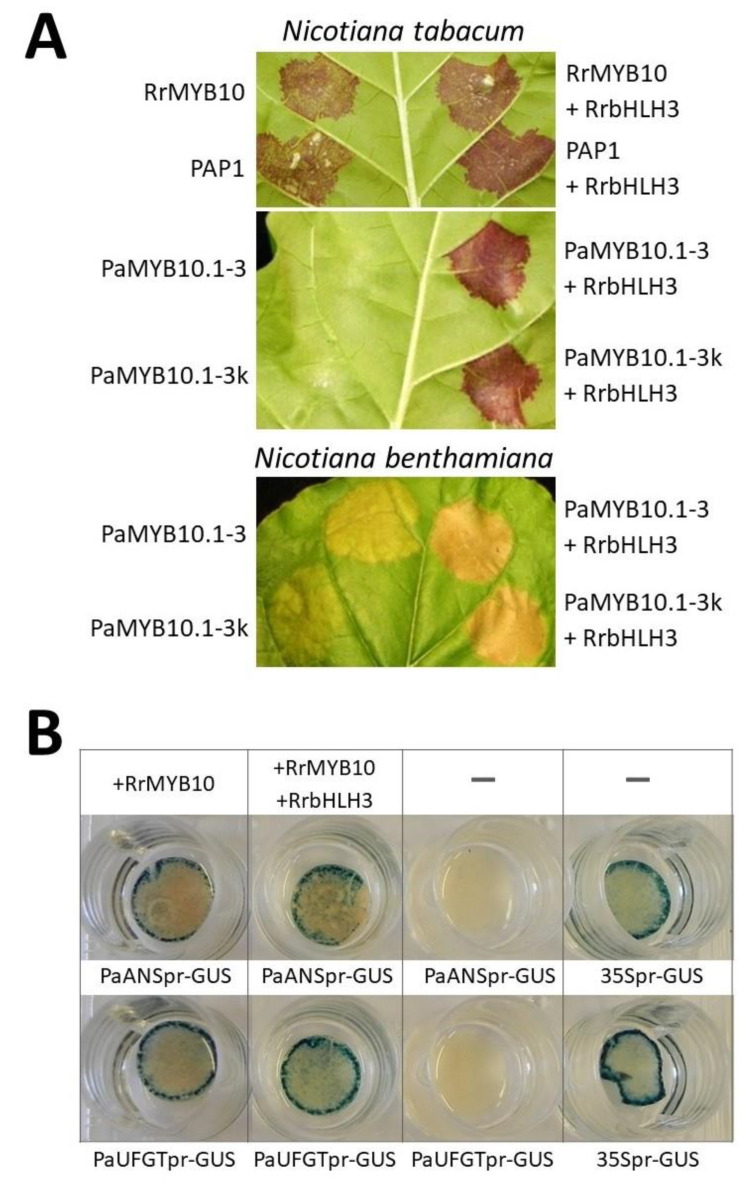
Transient expression of *Ribes rubrum* anthocyanin synthesis regulators in leaves of *N. tabacum* and *N. benthamiana* species. (**A**) The induction of anthocyanin synthesis by infiltrated regulatory genes. The comparison of the inducing capabilities of RrMYB10 and AtPAP1, and the effect of simultaneous infiltration of RrbHLH3 (top). The effect on the inducing capabilities of PaMYB10.1-3 elicited by the infiltration of RrbHLH3 in *N. tabacum* (middle) and *N. benthamiana* (bottom) leaves. PaMYB10.1-3k denotes an expression vector for cDNA of the PaMYB10.1-3 gene. (**B**) The reporter activation assay in *N. tabacum* leaves. GUS (β-glucuronidase) expression cassette under the control of *P. avium* UFGT or ANS promoter was infiltrated together with RrMYB10 or both RrMYB10 and RrbHLH3. Single GUS expression cassette under the control of PaUFGT or PaANS promoter served as a negative control, whereas single GUS expression cassette under the control of cauliflower mosaic virus 35S promoter served as a positive control.

The presence of specific domains and motifs, overall sequence homology to their counterparts in other species (Figure 3 and Figure 4) and the expression pattern (Figure 5A,B) of cloned *MYB10* genes in cultivars with varying anthocyanin levels suggest that they are functional regulators of the anthocyanin biosynthesis pathway. However, the ability to induce or enhance anthocyanin synthesis in some experimental system could show substantial proof of their function. Gene overexpression in *Nicotiana tabacum* leaves is one such system. Tobacco leaves possess functional genes of the anthocyanin pathway and, probably, a sufficient level of WD40 protein, because no significant differences of its expression are observed between green leaves and anthocyanin-accumulating transgenic tobacco leaves [31], and the addition of a heterologous *WD40* gene also did not observably influence anthocyanin accumulation induced by heterologous *MYB* and *bHLH* genes in previous infiltration experiments [32]. Thus, the expression of relevant MYB and bHLH proteins from infiltrated heterologous genes can lead to the formation of an active MBW complex, which can upregulate native tobacco anthocyanin pathway genes. We analyzed the ability of *RrbHLH3* and *RrMYB10* genes to induce anthocyanin synthesis while transiently expressed in two *Nicotiana* species. *RrMYB10* induced clear red patches after infiltration into *N. tabacum* leaves even without the simultaneous infiltration of any bHLH gene (Figure 6A, top left).

To test the ability of *R. rubrum* bHLH3 to assist MYB proteins in inducing anthocyanin synthesis, *RrbHLH3* was infiltrated together with *RrMYB10* or *A. thaliana* MYB10-like regulator *PAP1*. In these experiments, the effect of *RrbHLH3* infiltration was hardly noticeable because *RrMYB10* and *PAP1* induce strong anthocyanin synthesis by themselves (Figure 6A, top right vs. top left). Thus, in a subsequent experiment, we infiltrated *RrbHLH3* together with another MYB, *P. avium* anthocyanin regulator *PaMYB10.1-3*. *PaMYB10.1-3* alone is unable to induce anthocyanin synthesis in *N. tabacum* and only moderate anthocyanin synthesis in *N. benthamiana*, but is able to induce strong anthocyanin synthesis in both *Nicotiana* plants when infiltrated together with the relevant *bHLH* gene. The results showed that *RrbHLH3* is able to function as a coactivator: in *N. tabacum*, such a pair of genes induced anthocyanin synthesis (Figure 6A, middle), whereas in *N. benthamiana*, anthocyanin synthesis was enhanced (Figure 6A, bottom). When infiltrated alone, *RrbHLH3* was unable to induce anthocyanin synthesis, like other tested bHLH genes.

To test the ability of *RrMYB10* and *RrbHLH3* genes to induce anthocyanin pathway components from the rosid clade, we cloned promoters of *P. avium ANS* and *UFGT* genes. The promoters were fused with the *Escherichia coli* β-glucuronidase coding sequence, and such expression vectors were infiltrated together with the analyzed genes into the same *N. tabacum* system. The results were the same as in the anthocyanin induction experiments: *RrMYB10* alone was able to activate *PaANS* and *PaUFGT* promoters, whereas supplemented *RrbHLH3* slightly enhanced this induction (Figure 6B).

## 3. Discussion

In this work, we cloned anthocyanin pathway genes and their regulators from several species of the *Ribes* genus. Since this genus, as well as the whole Saxifragales clade, has not been extensively analyzed at the molecular biology level, the sequence information itself presents scientific value. In general, anthocyanin pathway enzymes show a high degree of homology with the counterparts from other species, especially at the amino acid level (Figure 2 and Figures S1–S9). The conservancy of the WD40 protein was also expected. This only constant member of the variable regulatory MBW complex has a highly conserved structure because it participates in the regulation of various processes in various tissues by not defining functional specificity of different complexes [33,34,35].

The second member of the regulatory complex can be any protein belonging to subgroup IIIf [36] of the bHLH protein family [37,38]. In *Arabidopsis*, these are the TT8, EGL3/GL3 and MYC1 proteins. The first member of the bHLH subgroup IIIf proteins is *Arabidopsis* TT8 and its homologs, named bHLH3 in most Rosaceae species, and An1 in tobacco (the branch named bHLH3 in Figure 3A). MBW complexes containing bHLH3-like proteins activate anthocyanin synthesis in plant tissues [39,40]. The functions of other subgroup IIIf bHLH proteins are less clear. Closely related proteins EGL3 and GL3 upregulate anthocyanin synthesis in *Arabidopsis*, but their homologs in other species, named JAF13 in tobacco and varyingly in other species (the branch named bHLH43 in Figure 3A), have not been studied extensively. According to recent data, tobacco JAF13 activates anthocyanin synthesis indirectly, by activating the expression of *NtAn1* [39]. The third bHLH protein, which can take part in the MBW complex, is *Arabidopsis* MYC1, named bHLH33 in most Rosaceae and some other species (the branch named bHLH33 in Figure 3A). This bHLH protein shows activating [41] or repressing [32] activities in different rosid species and is absent in the asterid clade. We cloned the bHLH3 protein as the most obvious activating bHLH member of the MBW complex.

The diversity of MYB proteins entering the MBW complex is large and embraces activators as well as repressors, proteins with different structures that are differentially expressed and active only in specific organs, tissues and developmental stages and those that regulate different branches of the flavonoid pathway [42,43]. Sequence analysis of cloned *MYB* genes (Figure 4) reveals that they encode R2R3 class subgroup 6 proteins [27]. Proteins belonging to this group there named MYB10 in Rosaceae [28,41] and in some other species [44,45]. It is well established that MYB10-like proteins activate the anthocyanin pathway in fruits [12]. The grouping of MYB10-like sequences belonging to the asterid, rosid and Saxifragales clades is not so strict, probably because there are differently specialized anthocyanin-inducing MYB10-like proteins in a single organism, whereas the phylogenetic tree is composed of arbitrary selected subgroup 6 sequences from each organism (Figure 4A).

Most flavonoid pathway enzymes in many plants are encoded by more than one gene, and different genes for the same enzyme can be differently regulated temporarily as well as spatially. Data generated by transcriptomic studies on anthocyanin synthesis regulation in bayberry, black raspberry and sweet cherry reveal that the expression of at least one gene encoding both late and early flavonoid pathway enzymes strongly correlates with anthocyanin level [16,17,18]. Expression analysis of anthocyanin pathway genes in fruits of different *Ribes* genus cultivars revealed that the activities of most enzyme-coding genes strongly correlate with fruit anthocyanin content (Figure 5). Thus, cloned *Ribes* cDNAs represent the enzyme genes responsible for anthocyanin accumulation in fruits, although there may be additional genes active in different tissues or causing the accumulation of flavonols in earlier stages.

In contrast to enzyme-coding genes, WD40 showed a similar expression level in all cultivars. This is in agreement with results obtained for *P. avium*, in which the expression of WD40 was almost constant in different cultivars and fruit maturation stages [32,46]. The expression of bHLH3 was more variable and to some degree correlated with anthocyanin content. In the fruits of other plants, bHLH3 expression is usually variable and in some plants positively correlates with anthocyanin production, such as in *Lycium ruthenicum* [47] and *Brassica oleracea* [48], but not in others, such as *Prunus avium* [32,46]. The expression of MYB10 strongly and reliably correlated with anthocyanin content in both *R. nigrum* and *R. uva-crispa*, but was not as high as in some other plants, where its level sometimes reached that of the highly expressed enzyme genes. The differences in the expression of MYB10 in the three cultivars of each species were not exactly replicated by the differences in expression of the late anthocyanin pathway genes ANS and UFGT. This may suggest that ANS and UFGT genes are also regulated by some other regulatory factors, not identified in this work.

Transient expression assays in tobacco leaves confirmed bHLH3 as a coactivator protein and revealed that RrMYB10 can induce anthocyanin synthesis without the addition of any helper bHLH. Although many anthocyanin-inducing MYB proteins require the addition of some external bHLH, some MYB10-like proteins, for example, MYB1 from lychee fruit [49] or MYB110 from kiwifruit petals [50], are also able to induce anthocyanin synthesis without any external bHLH proteins. In the latter case, an external MYB protein seems to be able to induce the expression of relevant tobacco bHLH proteins [39]. Thus, the induction of anthocyanin synthesis in *N. tabacum* without any helper bHLH proteins reveals the strong activating capabilities of RrMYB10 and suggests its ability to induce the expression of endogenous tobacco bHLH genes.

Sequence information obtained in this work can be applied in *Ribes* breeding programs and further anthocyanin research. With gene sequences of anthocyanin pathway enzymes and their main regulators available, it is possible to search for the exact sequence differences responsible for different anthocyanin levels and composition in various cultivars. This can accelerate the development of varieties with desired traits by applying molecular genetics methods to conventional breeding or by the use of genetic engineering. With verified qPCR primers available, which are suitable for the analysis of most important *Ribes* species, molecular methods can be used to analyze gene expression in response to various environmental stimuli and other factors. The analyzed anthocyanin regulators, especially *RrMYB10*, which has strong anthocyanin-inducing capabilities, can be exploited in manipulating anthocyanin synthesis in heterologous plant systems.

## 4. Materials and Methods

### 4.1. Plant Material

Fully mature fruits, which met the criteria of commercial ripeness defined individually for each cultivar (relevant color, size and taste), were collected from *R. nigrum* cultivars “Ben Tirran”, “Veera” and clone “79-197-7”; *R. uva-crispa* cultivars “Chorny negus”, “Lūšiai” and “Kursu dzintars”; *R. rubrum* cultivar “Jonkheer van Tets”, and *R. aureum* cultivar “Corona’”. Samples representing each cultivar consisted of twenty berries collected from five different plants. Bushes were grown at the Institute of Horticulture of Lithuanian Research Centre for Agriculture and Forestry, Babtai under field conditions (lat: 55.0000, long: 23.8000; elevation: 120 m). After harvesting, fruits were immediately frozen in liquid nitrogen and stored at −80 °C for subsequent analysis. *N. tabacum* and *N. benthamiana* were used for transient transformation experiments. *Prunus avium* cultivars “Irema BS” and “Kitayanka” were used for the isolation of promoter sequences. Measurement of the total anthocyanin concentration was performed as described earlier [32].

### 4.2. DNA Isolation, PCR, Gene Cloning and Sequencing

DNA for gene cloning was isolated from shoots of *R. nigrum*, *R. uva-crispa*, *R. aureum* and *P. avium* plants using a GeneJET Plant Genomic DNA Purification Mini Kit (Thermo Fisher Scientific, Lithuania, Vilnius). Gene fragments were isolated by PCR using partially degenerated primers designed to match the consensus sequence derived by aligning homologous sequences from the Pentapetalae taxonomic branch (Table S1). Sequences for the alignments were obtained by analyzing NCBI nucleic acid (NR), transcript shotgun assembly (TSA) and whole genome sequence (WGS) databases. PCR reactions were performed with Phusion High-Fidelity DNA Polymerase (Thermo Fisher Scientific, Lithuania, Vilnius). All PCR fragments were cloned and sequenced.

### 4.3. Isolation of RNA, cDNA Synthesis and RACE

Pieces of 3–5 berries containing 0.5 g of mesocarp and exocarp tissue from each *Ribes* cultivar were finely ground in liquid nitrogen and isolated with a Sigma Spectrum Plant Total RNA Kit. The cDNA synthesis was performed with a RevertAid H Minus First Strand cDNA Synthesis Kit (Thermo Fisher Scientific, Lithuania, Vilnius). For qPCR analysis, RNA samples were treated with RNase-free DNase I before cDNA synthesis. The 3′- and 5′-RACE were performed to obtain sequence information for regions outside those cloned with degenerated primers and to obtain full coding frames of genes used in functional experiments. For 3′-RACE, cDNA was synthesized using primer 3-RACE-CDS-A. For 5′-RACE and qPCR, cDNA was synthesized using polyT primer. A template-switching reaction for 5′-RACE was performed with SMART-C3 oligo using Thermo Fisher Scientific enzymes and buffers, similarly as described previously [51]. Direct and nested RACE PCR was performed using a gene-specific primer together with primer mix UPM and primer NUP-A, respectively. All primer sequences for RACE have been published previously [32]. All obtained sequences were submitted to the European Nucleotide Archive (ENA) under submission numbers LN736309–LN736346.

### 4.4. Gene Expression Analysis

All qPCR assays were performed according to MIQE Guidelines [52]. A Rotor-Gene Q system (Qiagen) was used to perform quantitative real time PCR (qPCR) amplification. All reactions were performed with Maxima SYBR Green/ROX qPCR Master Mix (Thermo Fisher Scientific, Lithuania, Vilnius) in a 20 μL volume with the following cycling parameters: 10 min at 95 °C, 40 cycles for 15 s at 95 °C and 60 s at 60 °C; melting curve detection from 60 °C to 95 °C. Three biological samples, each composed of five berries, were analyzed, and three technical replications were performed. Data were analyzed with Rotor-Gene Q Series software version 2.1.0 using the ΔΔCt method [53]. The expression level was normalized to *Ribes* actin (ENA LN736321). The qPCR reaction products were verified by melting curve analysis, electrophoresis and sequencing. All qPCR primer sequences are listed in Table S2.

### 4.5. Construction of Plasmids for Transient Gene Expression Assays

The *RrMYB10* and *RrbHLH3* genes, as well as ~1.5 kb fragments of both *Prunus avium* promoters (*PaANS* and *PaUFGT*), were PCR amplified with Phusion polymerase and cloned into a pJET1.2 vector. NruI/NotI-digested *RrMYB10* and *RrbHLH* were recloned into SmiI/NotI-digested vector pENTR/D (Thermo Fisher Scientific, Lithuania, Vilnius). Finally, the *RrMYB10* and *RrbHLH* genes were inserted into a destination vector pAUGLR by recombination using Gateway LR clonase II enzyme mix (Thermo Fisher Scientific, Lithuania, Vilnius). EcoRV/SalI-digested *PaANS* and EcoRI/XhoI-digested *PaUFGT* promoter fragments were cloned into Ecl136II/XhoI-digested vector pAUGLR. A gene encoding *E. coli* β-glucuronidase was inserted downstream of the cloned promoters by recombination using Gateway LR clonase II enzyme mix. Primers used for gene cloning and the construction of expression vectors are provided in Table S3. Plasmids for the expression of *AtPAP1*, *PaMYB10.1-3* and *PaMYB10.1-3k* were constructed as described previously [32]. For infiltration experiments, all final plasmids were electroporated into *Agrobacterium tumefaciens* GV3101 strain.

### 4.6. Transient Gene Expression Assays and Plant Transformation

*N. tabacum* and *N. benthamiana* plants were grown in soil at 25 °C in a room with controlled environment under long day conditions of 16 h of white light and 8 h of dark. An *A. tumefaciens* suspension was syringe infiltrated into the abaxial side of the leaves of five- to six-week-old plants. Overnight cultures of *A. tumefaciens* were grown in LB medium with rifampicin and spectinomycin, diluted 500-fold with LB containing 10 mM MES (2-(N-morpholino)ethanesulfonic acid), and grown overnight till A600 = 1.2–1.6. After sedimentation by centrifugation for 20 min at 3000 ×*g*, the cells were suspended in infiltration buffer (10 mM MES pH 5.7, 10 mM MgCl2, 500 µM acetosyringone) till A600 = 1.8 and incubated overnight at room temperature.

A helper strain containing a silencing suppressor from plum pox virus (PPV HC-Pro) [54] was used in all infiltration experiments. When testing several expression cassettes in a single infiltration, transformed *Agrobacterium* strains were mixed at equal ratios. Coloration around infiltration zones, indicating anthocyanin synthesis, was observable on the abaxial side of *N. tabacum* leaves and the adaxial side of *N. benthamiana* leaves starting from three days post infiltration. Photographs were always taken seven days post infiltration. In all experiments, the expression of infiltrated genes was verified by RT-PCR.

### 4.7. Promoter Activity Assays Using Reporter Gene Expression

Promoter activity assays were performed by infiltrating promoter::GUS plasmids into *N. tabacum* leaves, as already described. Leaf discs with the infiltration point at the center were cut out using a 12 mm diameter cork borer thirteen days post infiltration. Isolated leaf discs were placed abaxial side up into a 24-well plate containing X-gluc buffer solution (0.25 mg/mL X-gluc (5-Bromo-4-chloro-1H-indol-3-yl β-d-glucopyranosiduronic acid) in DMSO, 0.5 mM potassium ferrocyanide (K4Fe(CN)6 3H2O), 0.5 mM potassium ferricyanide (K3[Fe(CN)6]), 0.01% Tween 20, 100 mM sodium phosphate buffer), and vacuum applied for 12 min. Samples were incubated in the dark at 37 °C for 24–48 h until the distinct blue staining appeared. Finally, leaf discs were washed several times in a series of 70% and 80% (*v*/*v*) aqueous ethanol solutions until the chlorophyll was removed.

### 4.8. Bioinformatics Analysis

Sequences of plant anthocyanin pathway genes were obtained by BLAST searching NCBI NR, WGS and TSA databases (http://www.ncbi.nlm.nih.gov). Multiple sequence alignments were accomplished with Clustal Omega [55], and phylogenetic trees were created with MEGA version 7 [56] using the neighbor-joining method with 1500 bootstrap repetitions. Sequence logos were created with WebLogo 3 [57]. Protein domains and amino acids with known functionality were annotated by NCBI’s conserved domain database (CDD) [58]; secondary structure was predicted using PSIPRED [59].

## Supplementary Materials

The following are available online at https://www.mdpi.com/2223-7747/9/9/1078/s1, Figure S1: Phylogenetic tree of selected UFGT proteins, Figure S2: Phylogenetic tree of selected ANS proteins, Figure S3: Phylogenetic tree of selected DFR proteins, Figure S4: Phylogenetic tree of selected F3H proteins, Figure S5: Phylogenetic tree of selected CHI proteins, Figure S6: Phylogenetic tree of selected CHS proteins, Figure S7: Phylogenetic tree of selected WD40 proteins, Figure S8: Phylogenetic tree of selected bHLH3 proteins, Figure S9: Phylogenetic tree of selected MYB10 proteins, Table S1: Partially degenerated primers for used to clone fragments of *Ribes* anthocyanin pathway genes, Table S2: qPCR primers used in the study, Table S3: Primers for construction of plasmids for transient gene expression and promoter cloning from the genome.
