# Supplementary material for: Isolation and Analysis of Anthocyanin Pathway Genes from Ribes Genus Reveals MYB Gene with Potent Anthocyanin-Inducing Capabilities
Source: Plants (Basel). 2020 Aug 22;9(9):1078. doi: 10.3390/plants9091078 (PMC7570362; doi:10.3390/plants9091078)
Supplement: Supplementary file 1 [file plants-09-01078-s001.pdf]

Table S1. Partially degenerated primers for used to clone fragments of *Ribes*anthocyanin pathway genes.

Name	Sequence (5′ → 3′)	Gene
**UDRR3d**	TGYTTGRTSASTGAYGCITTCYTTTGGTT	UFGT
**UDRR6r**	CCAAAAAADGGCCTRCAAATCATDGGYAC	
**ANSR3d**	TGGGGTGTBATGCACCTKGTBAAYCAYGG	ANS
**ANSR8r**	GGCTTVAGGATRATCTTCTCCTTNGGYGGYTC	
**DFR1d**	GGDTTCATYGGBTCWTGGCTYRTCATG	DFR
**DFR10r**	ACATGTCCTCYAAGYTGTAYTTGAAYTBRAAYCC	
**F3H2d**	CATCGTCTCCAGCCATTTACAGGGRG	F3H
**F3H5r**	CWGGTTGAACGGTGATCCACGTC	
**CHIR1d**	CCAASACYYTSTTYCTCGGYGGYGCAGG	CHI
**CHIR3r**	AGMACWGCCTCSGAMAGYWGYTTRTTYTC	
**CHSR1d**	CCTGAYTACTACTTTCGTATCAC	CHS
**CHSR2r**	ACGCTRTGMARCACMACGGTCTC	
**WDR1d**	TCBCCBTACCCDMTBTACKCCATG	WD40
**WDR15r**	ATCCAATCMGGCTGHGCAGCAGACCAYTG	
**HLHR12d**	TGGTTYTAYYTSATGTGYGTYTCHTTCTC	bHLH3
**HLHR16r**	GCTCTDGARAARRYTTTGCTRTCRACYTCRTTTG	
**MDGR1d**	GAAAAGGTGCHTGGACYVIAGARGAAGA	MYB10
**TaR2r**	GTGTTCCARTARTTYTTCACATCRTTNGC	

Table S2. qPCR primers used in the study.

Primer	Sequence (5′ → 3′)	Gene
**Forward**	TGGCCCTGCTATCTTGGATC	CHS
**Reverse**	ACTTCCTCCTCATCTCGTCC	
**Forward**	AGAGTGCAGAAGAGTTGACGG	CHI
**Reverse**	TCCAACAGACTTCCAAAAGGC	
**Forward**	GAATGCGGATCACCAAGCAG	F3H
**Reverse**	AGCTCAATGTCTTTGCTCATCTTC	
**Forward**	CGGACTTTCGCCAATCACTC	DFR
**Reverse**	GCAGAGCAAATGTAGCGTCC	
**Forward**	GCACTCACCTTCATACTCCAC	ANS
**Reverse**	TGCTCTTGTACTTGCCATTGC	
**Forward**	GTGGCTGGAACTCATTGCTG	UFGT
**Reverse**	CTCCAAAATGTCCTCCACCAC	
**Forward**	CGAGAGTCCACAACCCGATAC	WD40
**Reverse**	CATTCACACTCGCCTTATGCC	
**Forward**	TTAGGATTGTGGAGGGGAGTG	bHLH3
**Reverse**	ACAACCCTTCTTGATGCTGAC	
**Forward**	GGCTGAAGAAGAAGTTGGAGGA	MYB10
**Reverse**	TGTCCATATCAAAGGAGGGAGAC	
**Forward**	CGACTATGTTCCCTGGTATTGC	Actin
**Reverse**	CCCTTGGAAATCCACATCTGC	

Table S3. Primers for construction of plasmids for transient gene expression and promoter cloning from the genome.

Name	Sequence	Gene
**P418 fwd NruI**	ATCGCGAATGGAGAGAGATTTTACGGA	RrMYB10
**P418 rev NotI**	TGCGGCCGCTCAGGCCACCATTTCTTTTT	
**P419 fwd NruI**	ATCGCGAATGGCTGCGCA	RrbHLH3
**P419 rev NotI**	AGCGGCCGCTTAATCTTGAGGTATAAT	

**P156 fwd EcoRV**	AGATATCCTTCTCCAATGTGCAGCTT	PaUFGT
**P156 rev XhoI**	ACTCGAGATATGTATGTGCTAATAAGACTAA	
**P83 rev SalI**	AGTCGACATTTTGGCAGCCG	PaANS
**P82 fwd EcoRV**	AGATATCGGATTGATTGTAAGTTTGAT	
**PrUFGTpr1d**	ATTCCCTGTCTTATCCTGTAATTTGG	PaUFGT, for cloning from the genome
**PrU_84-61r**	GGCATGGGTGGAGAAAGGGAAGGC	
**PrU_177-152r**	GAGTGAGTTGTTGGATTGTGAAGTGC	
**PrANSPr1d**	CTTCTGCCGAGTCTTCCCTCCAG	PaANS, forcloningfromthegenome
**PrANS3r**	CCACTGCTGGCCAAGGTCTCAAC	
**PrANS4r**	CGTTCTCAGAGTCTATCTCCTTCAAATC	

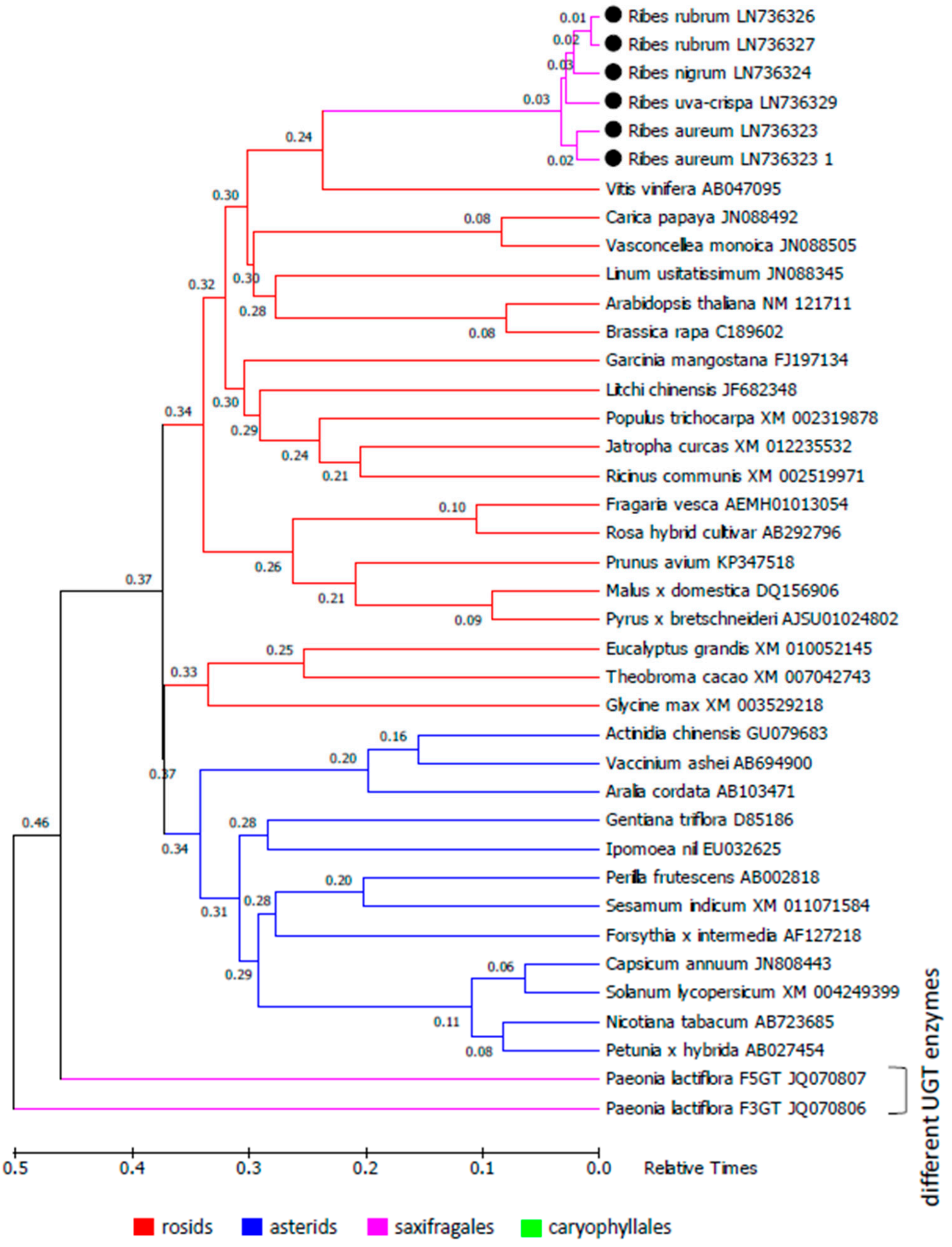

Figure S1. Phylogenetic tree of selected UFGTproteins.

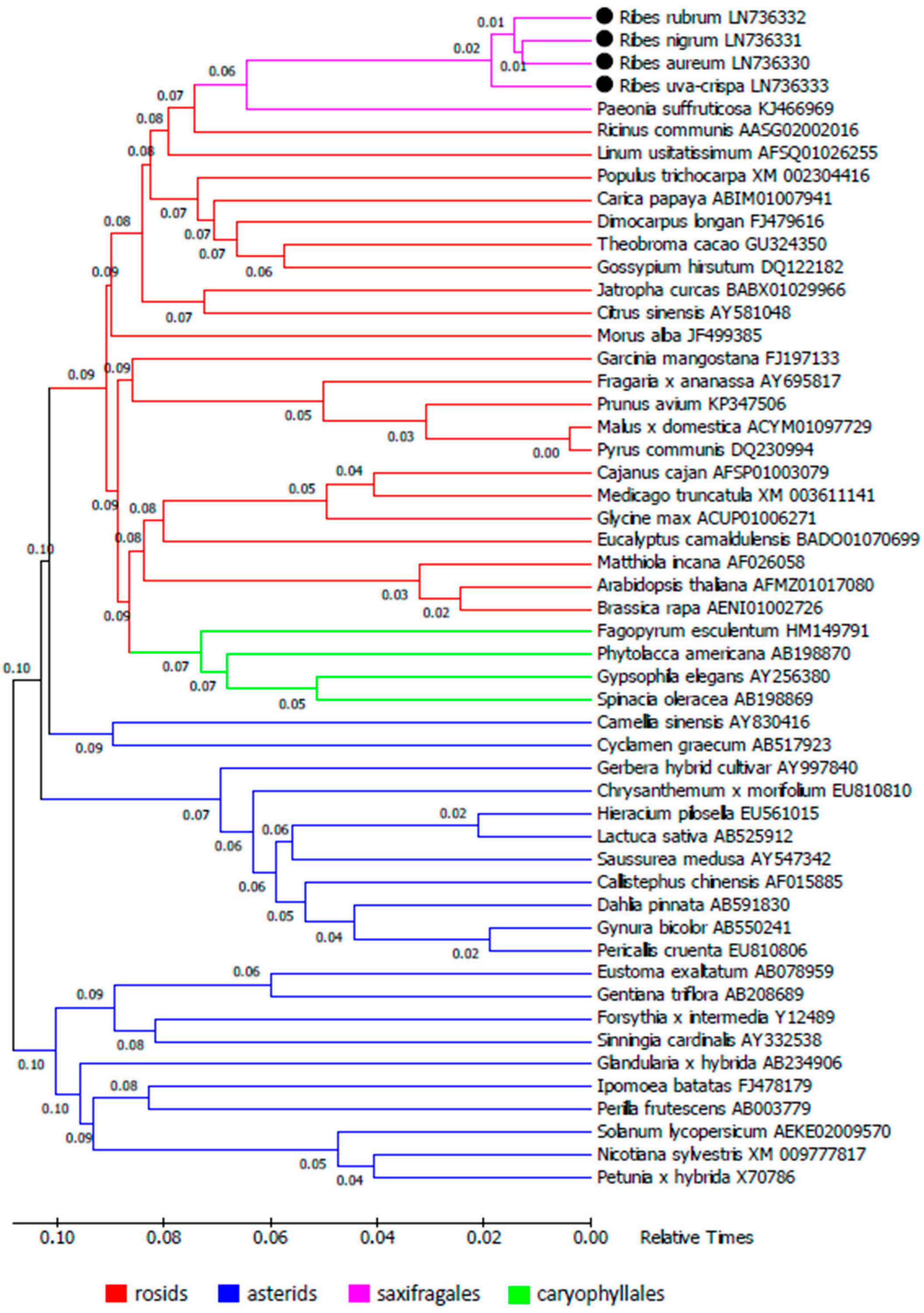

Figure S2. Phylogenetic tree of selected ANS proteins.

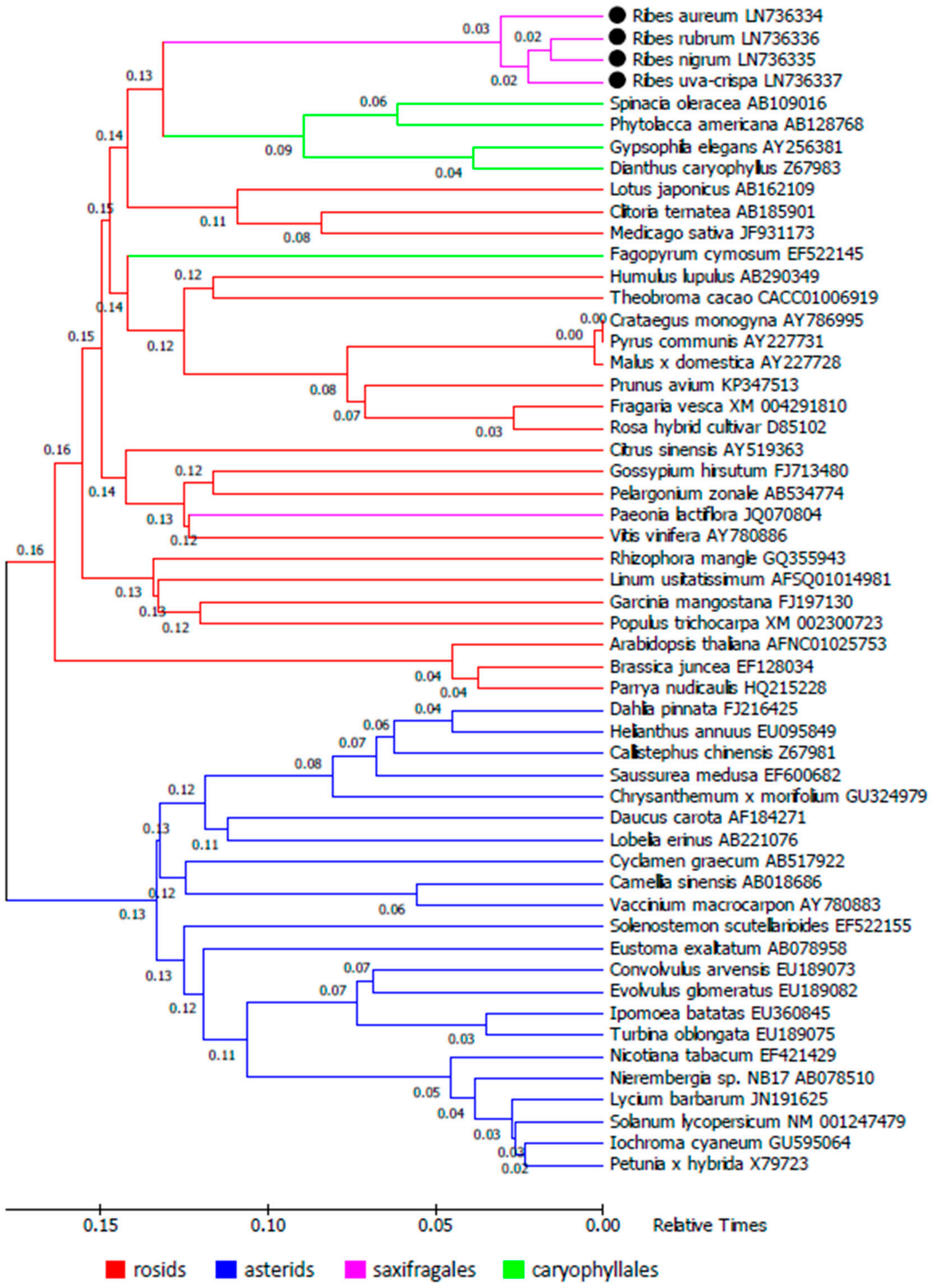

Figure S3. Phylogenetic tree of selected DFR proteins.

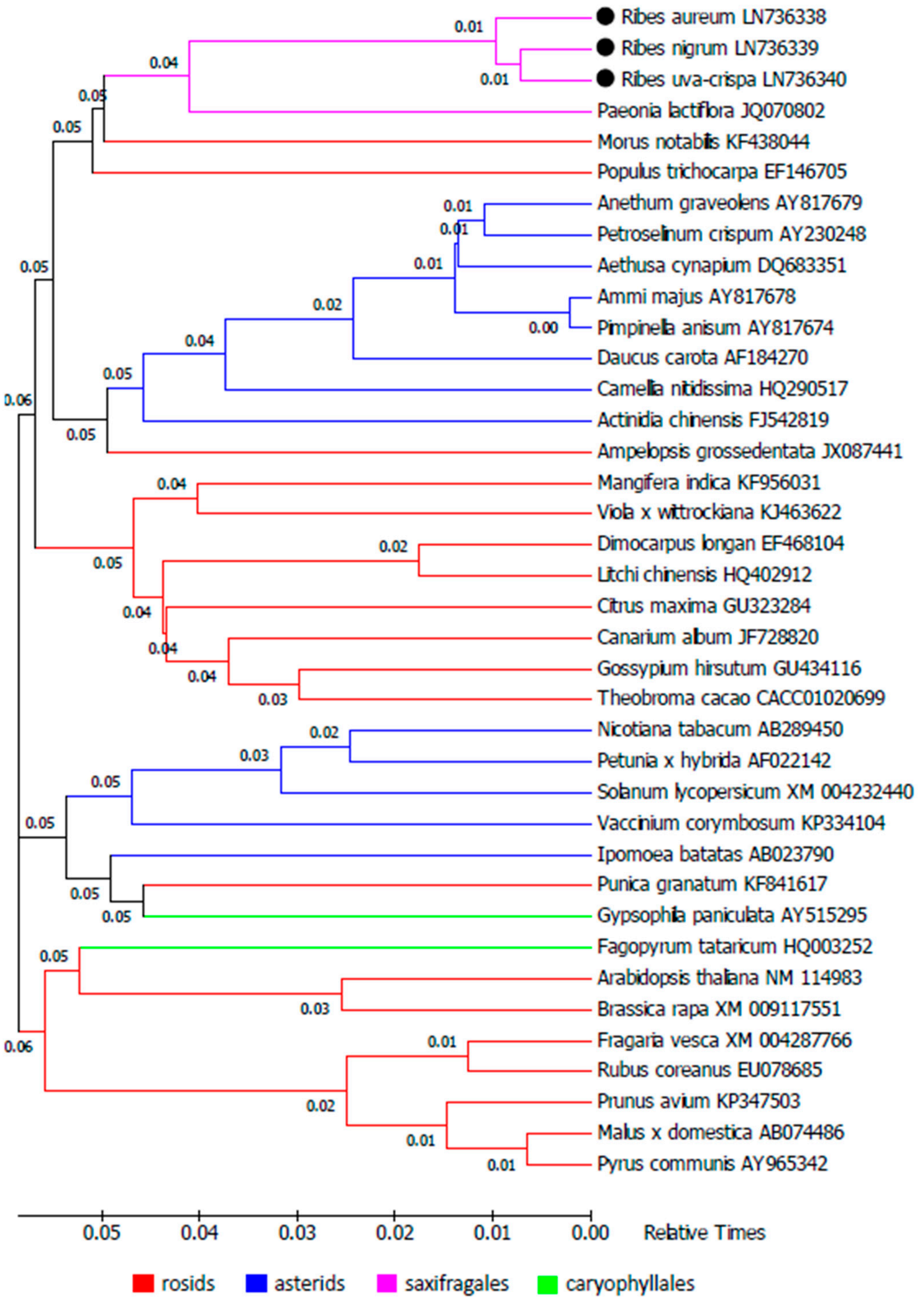

Figure S4. Phylogenetic tree of selected F3H proteins.

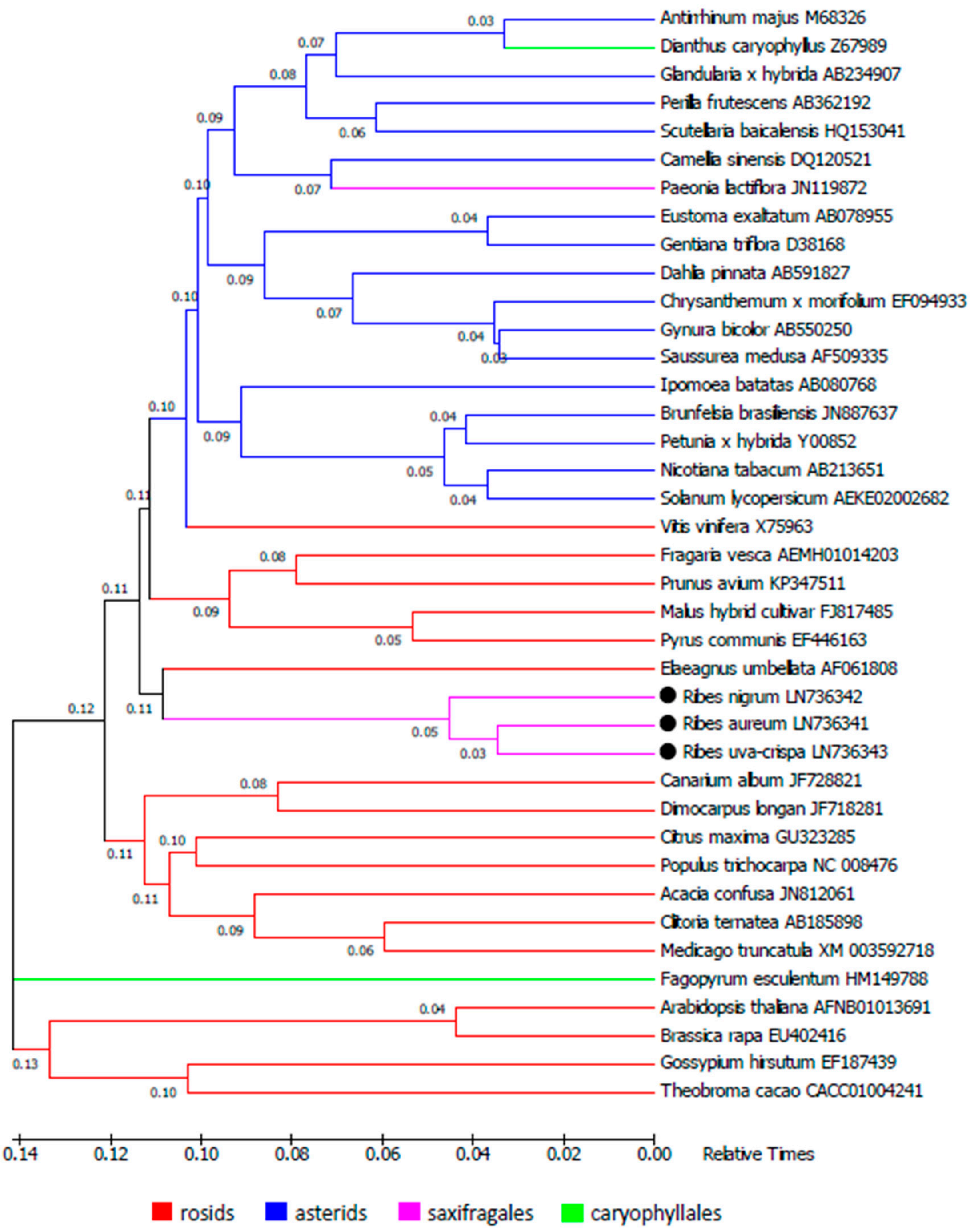

Figure S5. Phylogenetic tree of selected CHI proteins.

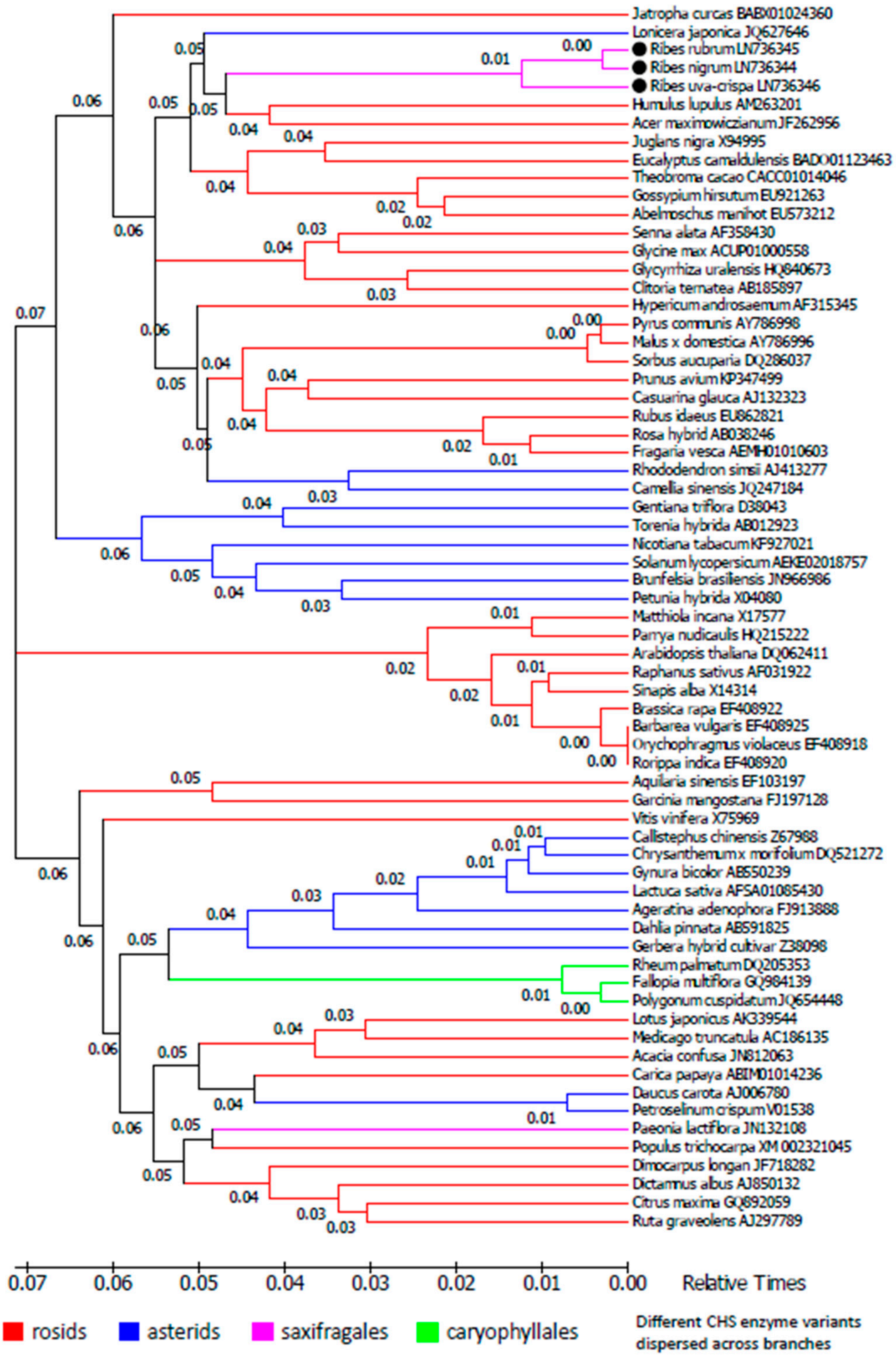

Figure S6. Phylogenetic tree of selected CHS proteins.

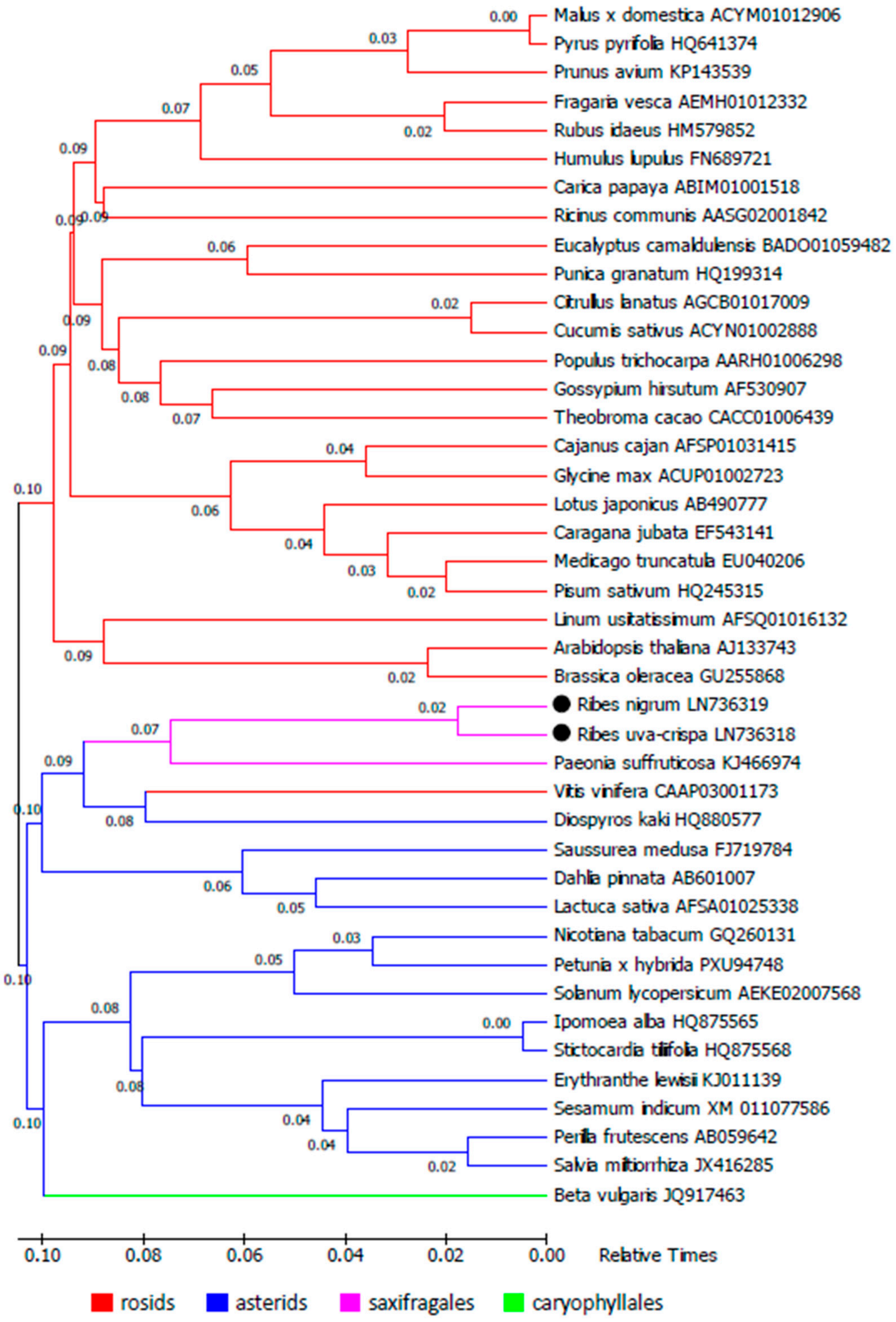

Figure S7. Phylogenetic tree of selected WD40 proteins.

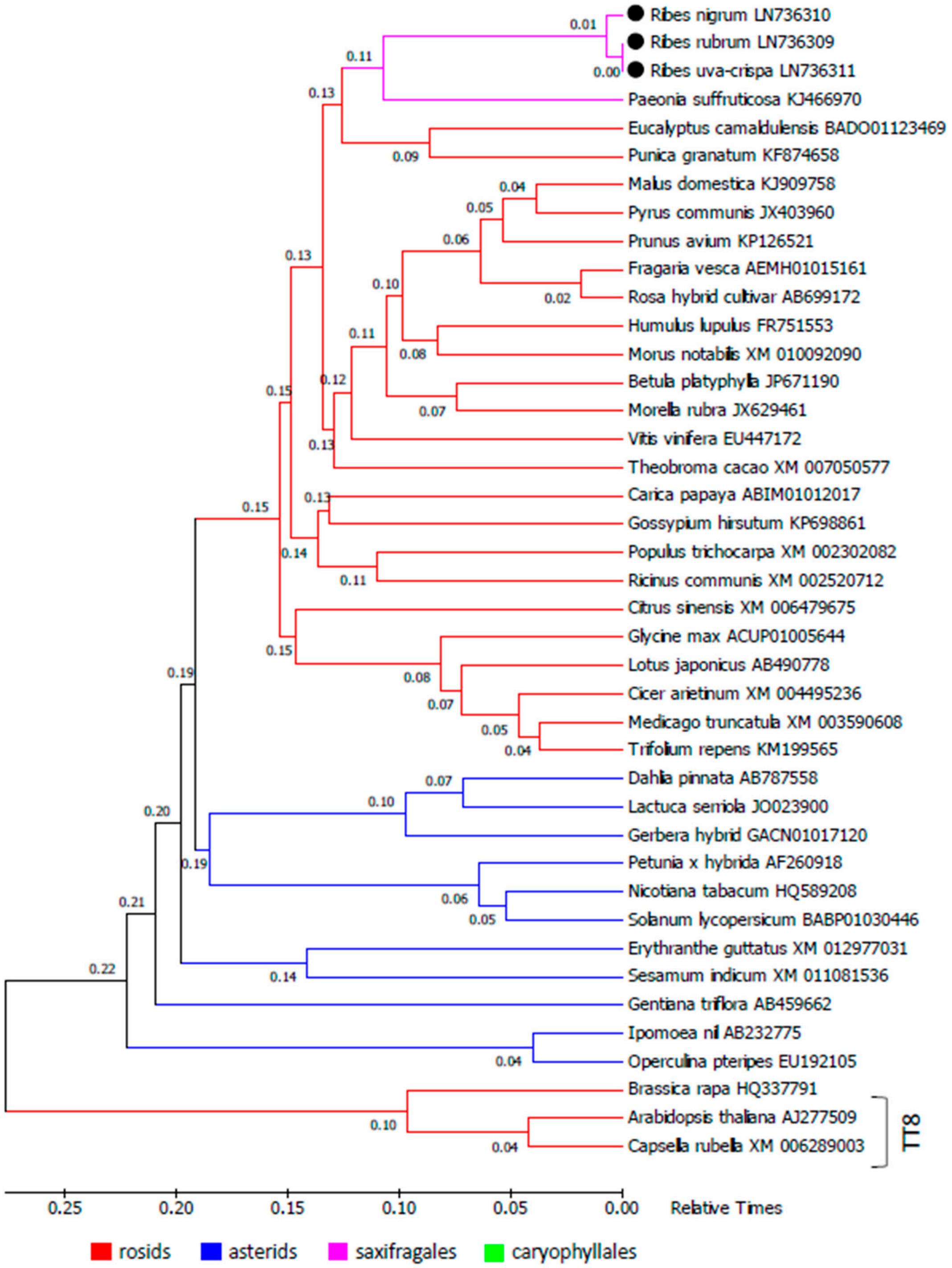

Figure S8. Phylogenetic tree of selected bHLH3 proteins.

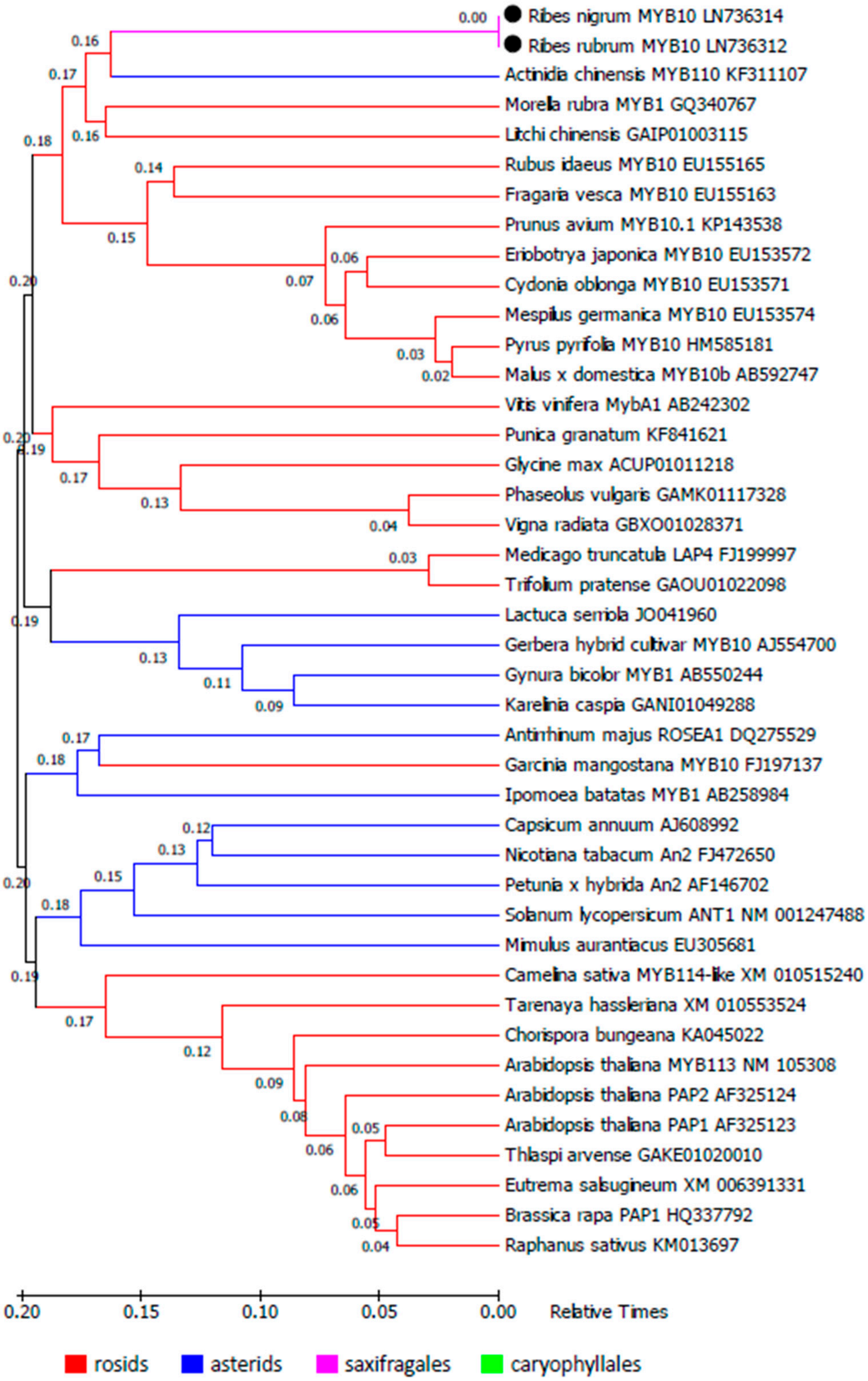

Figure S9. Phylogenetic tree of selected MYB10 proteins.
